# Assessment of radioactivity levels in shore sediments along the coastline of the Orange River, Oranjemund, Namibia

**DOI:** 10.1016/j.heliyon.2022.e10579

**Published:** 2022-09-11

**Authors:** Sylvanus Ameh Onjefu, Aina Nancy Iyaloo Kauluma, Munyaradzi Zivuku, Emmanuel Ejembi, Roswita Hambeleleni Hamunyela, Bismark Mzubanzi Tyobeka

**Affiliations:** a Department of Natural and Applied Sciences, Namibia University of Science and Technology, Windhoek, Namibia; b Department of Radiography, School of Allied Health Sciences, Hage Geingob Campus, University of Namibia, Windhoek, Namibia; c Office of the Vice Chancellor, North-West University, Potchefroom, South Africa

**Keywords:** Excess lifetime cancer risk, Radiological risk assessment, Shore sediments, Orange river

## Abstract

The objective of present study was to evaluate the activity concentrations of ^238^U, ^232^Th and ^40^K and radiological hazards due to gamma exposure of shore sediment along the coastline of the Orange River, Oranjemund, Namibia. A total of 20 shore sediment samples were collected along the coastline of the Orange River. Shore sediment samples were analyzed using a Canberra Gamma Spectrometric detector inter phased with a multichannel analyzer (MCA) that was well calibrated for energy and efficiency respectively. The mean activity concentrations of ^238^U, ^232^Th and ^40^K for the shore sediment samples were 63.46 ± 9.83 Bqkg^−1^, 54.88 ± 5.03 Bqkg^−1^ and 416.99 ± 57.85 Bqkg^−1^ respectively. The mean activity concentrations of ^238^U, ^232^Th and ^40^K in the shore sediment samples were slightly higher than world reference levels. Also, the radiological hazards parameters of absorbed dose rates, annual effective dose equivalent (outdoor) and the excess lifetime cancer risk for the Orange River shore sediment samples were calculated. The mean values calculated for absorbed dose rates (63.98 nGy/h), annual effective dose equivalent (outdoor) (0.78 mSv/y) and excess lifetime cancer risk (2.73) were higher than the recommended limits, therefore long term radiation exposure of the local population along the coastline of the Orange River may pose significant health threat from radiological point of view.

## 1 Introduction

Radiation is ubiquitously present in the environment due to natural sources such as radionuclides from terrestrial origin found in rocks, soil, food and water and radionuclides from the cosmic sources which are as a result of bombardment of heavy nuclei with other particles in the atmosphere (Orosun et al., 2021). Terrestrial radionuclides varies in spatial and temporal which is governed by the prevailing climatic conditions and the geology of the location, which influences the type of rock and soil (UNSCEAR, 2000; Onjefu et al., 2021a). Exposures to terrestrial gamma rays and to cosmic rays are the main components of an individual radiation doses received in the biosphere (Lilley, 2001).

The exposure to human population to elevated levels of ionizing radiation is harmful and can lead to cancer and other sicknesses caused by radiation (UNSCEAR, 2000; Orosun, 2016). Studies have shown that the increased levels of natural radioactivity in the environment are caused by both natural processed and human activities (Garba et al., 2008; Cember and Johnson, 2009; Taskin et al., 2009). For example, when rocks disintegrate through natural processes, radionuclides are released and carried away to soil, rivers, sediments and ocean by rain and flows (Taskin et al., 2009). Also, human activities such as mining, agricultural activities and atomic bomb testing also affect the levels of natural radioactivity in the environment (Oluyide et al., 2019; Amwaalanga et al., 2019; Onjefu et al., 2021b).

There has been a growing public concern in areas, which has been polluted by radioactive material due to chemical toxicity of uranium (Choy et al., 2006; ICRP, 1991; Miller and McClain, 2007). These radionuclides may be leached in the soil or transported to water bodies where they ultimately sinks and then incorporated into sediments where they may present a health risk to the human population.

In Namibia, the measurements of the activity concentrations of ^238^U, ^232^Th and ^40^K in different environmental matrices have been reported (Steinhausler and Lettner, 1992; Oyedele et al., 2010; Onjefu et al., 2017; Amwaalanga et al., 2019; Zivuku et al., 2018; Onjefu et al., 2021a, Onjefu et al., 2021b). For example, the study undertaken by Steinhausler and Lettner (1992) and Onjefu et al. (2017) showed that some regions of Namibia have high background radiation. Also, the study by Amwaalanga et al. (2019) indicated slightly high levels of natural radioactivity in river sediments which may be attributed to the use of agrochemicals such as fertilizers for agricultural practices along the bank of the Zambezi River at Katima Mulilo.

As one important economic and ecological zones on the Earth, shores of coastlines are a significant place of leisure and agricultural activities. Shore sediments also serve as habitat to crabs, bivalves and other rare marine organisms (Xinming and Wuhui, 2018). The levels of ^238^U, ^232^Th and ^40^K in sediments along coastline from different parts of the world have been well-documented (Abdi et al., 2008; Akram et al., 2006, 2007; Alam et al., 1999; Omeje et al., 2021; Khandaker et al., 2018; Xinming and Wuhui, 2018). However, only the study undertaken by Onjefu et al. (2017) and Amwaalanga et al. (2019) looked at naturally occurring radionuclides in shore sediments in Namibia even though the study reported by Onjefu et al. (2017) showed elevated levels of natural radioactivity in shore sediments along the coastline of the Erongo region of Namibia.

The objective of this present study is to assess the levels of natural radioactivity in the shore sediments of the Orange River, and evaluate the radiological hazards along the coastline. The findings from this study will serve as radiological database for the region and for future planning purposes regarding radiological mapping of the region.

## 2 Materials and methods

### 2.1 Study area

**Figure 1:**
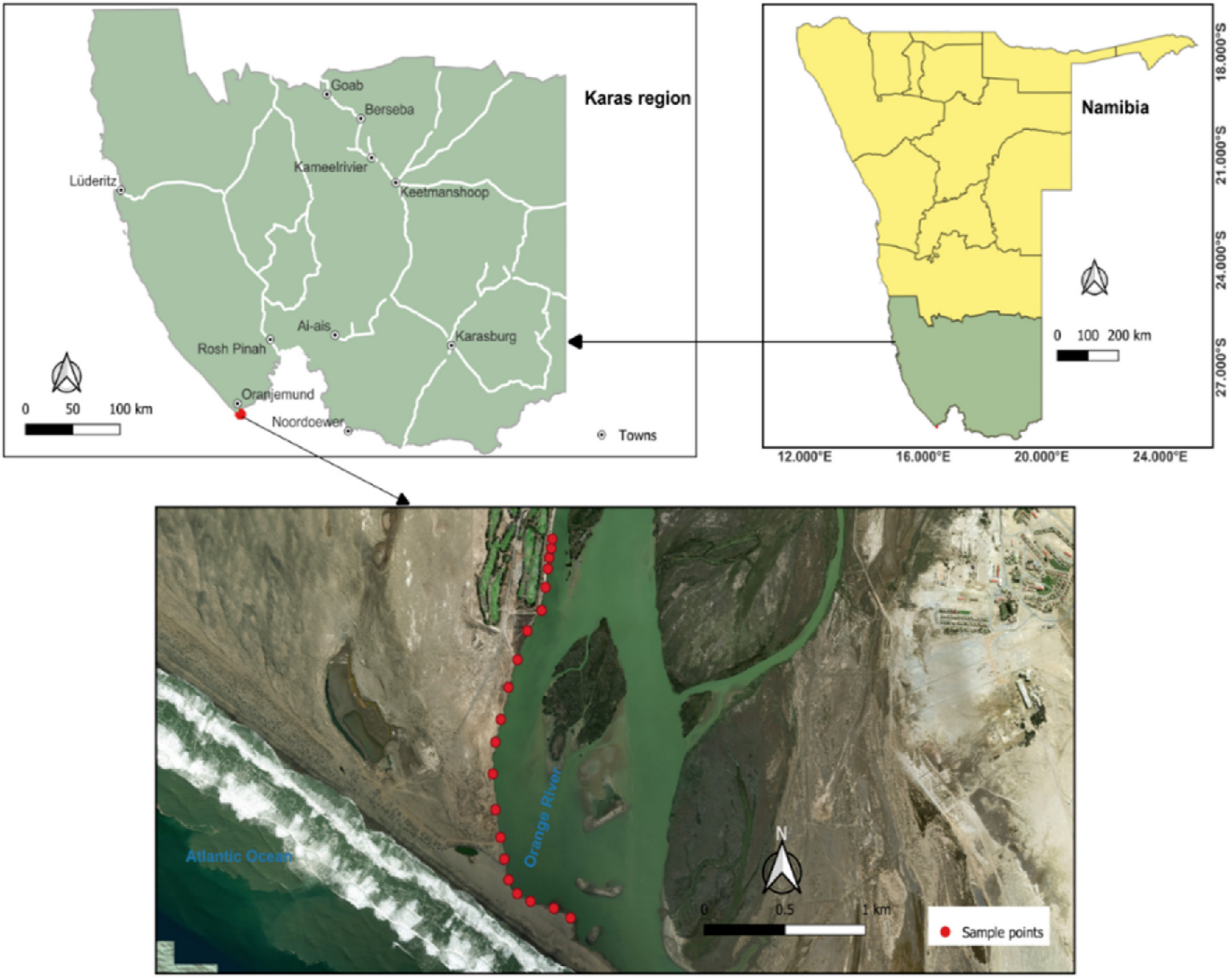
Study area and distribution of river sediment sample at Orange River, Oranjemund.

The Orange River is Southern Africa’s longest waterway. River Basin extends extensively into South Africa, Namibia, and Botswana to the north and its size is approximately 973 000 km^2^. The river has a total length of 2,200 km. The present study was undertaken along the coastline of the Orange River at Oranjemund and it is situated at Latitude – 28^O^37’59.99’’S and Longitude 16^O^26’59.99’’E (Figure 1). The geological setting of the Orange River at Orajemund is characterized by a distinctive cutting through the Mesoproterozoic Namaqua metamorphic complex (Thomas et al., 1994; Jacobs et al., 2008) and existing through the Neoproterozoic Gariep Belt (Frimmel et al., 2004) at the mouth of the river joining the Atlantic Ocean. There are a number of human activities along the bank of the Orange River at Oranjemund. Some of these activities includes mining activities, crops and small life stocks farming.

### 2.2 Sample collection and preparation

In this study, primordial radionuclides were measured in shore sediments samples using a high-purity germanium (HPGe) detector. About 1000 g samples were collected from a distance of 12 m parallel to the shoreline along the coastline of the Orange River using purposive stratified random sampling. A total of twenty (20) samples were collected from 11 cm depths because the vertical heterogeneity may represent many years of shore sediment changes. The samples were kept in cleaned and numbered polyethylene bags and all the sampling points from the identified sampling sites were marked by means of a Global Positioning System device (GPS) with model GPSMAP 62S and serial number IPH-01699. The collected samples were oven dried at 120 degrees Celsius for 12 h to dry off all the moisture. The samples were thoroughly mixed, ground and homogenised. About 500 ± 0.001 g of the homogenised samples were carefully packed in well labelled 500 ml beakers, sealed hermetically and kept for 30 days for the samples to achieve secular equilibrium (Onjefu et al., 2017).

The radioactivity in the collected samples were measured using a coaxial (62.80 × 64.80 mm) Canberra gamma-ray spectrometer HPGe detector Model No. GC4520 SN 10882 with 45% relative efficiency and resolution of 2.00 keV (FWHM) at 1.33 MeV peak of Co-60 and 1.200 keV (FWHM) at 122 keV. The detector has end cap outside diameter of 3.25 “Dia with a front window of 1 mm thick Al and end cap length of 5.25 “length. To minimize the effect of scattered radiation and avert increase environmental count, from the shield, detector is situated in the middle of the chamber.

The gamma spectrometry was calibrated for energy and efficiency using mixed radionuclides standard with a wide a range of gamma-ray energies 0.060 MeV–2 MeV in a 500ml beaker. These standards were supplied by Eckert & Ziegler Nuclitec GmbH, Germany, SN. AM 5599 and validated using IAEA NORMs reference material RGK-1, RGTh-1 and RGU-1. A computer based Multichannel Analyser (MCA) Genie 2000 software from Canberra was used for data acquisition and analysis of gamma spectra which identified background radiation from the recognized nuclides. The samples were counted for 43200 s with background count subtracted from the net count**.** For quality control, calibration for energy and efficiency was done in order to maintain quality of the measurements. The 295.22 keV, 351.93 keV for ^214^Pb and 609.32 keV, 1120.29 keV and 1764.49 keV for ^214^Bi gamma lines were used in the assessment of activity concentration of ^238^U, while 911.21 keV for ^228^Ac, 968.97 keV and 238.63 keV for ^212^Pb were used for ^232^Th. The single 1460 keV Gamma-line of ^40^K was used in its content evaluation.

The activity concentration of individual radionuclides in all the river sediment samples investigated was calculated using the following expression in Eq. (1) (Amin et al., 2013).

(1) $$A\left(Bqkg^{-1}\right)=\frac{N}{\in_{\gamma }P_{\gamma }T_{S}M}$$

where A is the specific activity in Bq/kg of each radionuclide in the sample, N is the net peak count rate of the resulting photo-peak, $\in_{\gamma }$ is the detector efficiency of the specific gamma-ray, $P_{\gamma }$ is the gamma emission probability of the corresponding gamma energy, $T_{S}$ is the counting time of the sample and M is the sample mass in kg. The error associated with every activity calculation was computed by the standard deviation Eq. (2) (Amin et al., 2013), derived from the uncertainty budget. The equal counting time for both background and sample was chosen to minimize the uncertainty in the net counts,

(2) $$\Delta A=\sqrt[{A}]{{\left(\frac{\Delta N}{N}\right)}^{2}+{\left(\frac{\Delta \in_{\gamma }}{\in_{\gamma }}\right)}^{2}+{\left(\frac{\Delta P_{\gamma }}{P_{\gamma }}\right)}^{2}+{\left(\frac{\Delta M}{M}\right)}^{2}+{\left(\frac{\Delta T_{S}}{T_{S}}\right)}^{2}}$$

where ΔA is the uncertainty of the sample measured and ΔN, $\Delta \in_{\gamma }$, $\Delta P_{\gamma }$, ΔM, and $\Delta T_{S}\;$ are the uncertainties of the net count rate, efficiency, gamma emission probability, sample weight, and counting time respectively.

### 2.3 Measurement of radiological hazard parameters

#### 2.3.1 Absorbed dose rate (ADR)

The absorbed dose rate is directly linked to the health risks related to human radioactivity exposure. ADR was calculated according to Eq. (3) (UNSCEAR, 2000; Beretka and Mathew, 1985).

(3) $$\text{ADR}\left({\text{nGyh}}^{-1}\right)=0.457{\text{C}}_{\text{U}}+0.6{\text{C}}_{\text{Th}}+0.042{\text{C}}_{\text{K}}$$

where C is the activity concentration of the primordial radionuclide in Bq/Kg.

#### 2.3.2 Annual effective dose equivalent (AEDE)

The absorbed dose rate does not provide us with enough information to estimate the human radiological risk, thus the conversion factor and the occupancy factors are taken into account to convert from absorbed dose to annual effective dose. For calculation purposes, a conversion factor of 0.7 Sv/Gy is used to convert the absorbed rate to a human effective dose with an outdoor occupancy of 20 % and 80 % for indoor (UNSCEAR, 2000). The annual effective dose equivalent was evaluated using Eq. (4) (Sivakumar et al., 2014):

(4) AEDE=D×T×F

where AEDE is the annual effective dose (mSv), D is the absorbed dose rate (nGy/h), T is the outdoor occupancy time (365 days × 24 h x 0.2) and F is the conversion factor 0.7 (x ($10^{3}$ mSv/nGy $10^{9}$)).

#### 2.3.3 Excess lifetime cancer risk (ELCR)

The excess lifetime cancer risk indicates the risk of death in a population brought on by cancer due to exposure to background radiation in excess resulting from a lifetime exposure to carcinogens. It indicates how many more additional cases of cancer one would expect in a population of people exposed to an excess amount of carcinogens for an average duration of life which is 70 years. The excess lifetime cancer risk is calculated according to Eq. (5) (Taskin et al., 2009):

(5) $$\text{ELCR}=\text{AEDE}\left(\text{out}\right)\;{\text{Sv ​y}}^{-1}\times \text{DL}\times {\text{RF ​Sv}}^{-1}$$

where AEDE (out) is the annual effective dose equivalent outdoors ${\text{Sv ​y}}^{-1}$, DL is the average duration of a lifetime which is 70 years, and RF is the risk factor (0.05 Sv^−1^). The risk factor is the fatal cancer risk per Sievert (Taskin et al., 2009).

## 3 Results and discussion

### 3.1 ^238^U, ^232^Th and ^40^K activity concentrations

The measured activity concentrations of ^238^U, ^232^Th and ^40^K and the calculated results of absorbed dose, annual effective dose equivalent and excess lifetime cancer risk for the 20-shore sediment samples are presented in Table 1. The mean activity concentrations were measured in Bq/kg and found to be 63.46 ± 9.85, 54.88± 5.03, and 416.99± 57.85 respectively in the order ^40^K > ^238^U > ^232^Th. Their maximum activity concentrations in Bq/kg were 114.27 ± 23.45 for ^238^U, 91.20 ± 9.22 for ^232^Th, and 473.37 ± 63.44 for ^40^K and their minimum activity concentrations were 33.67 ± 23.45 (^238^U), 37.15 ± 3.03 (^232^Th), and 330.39 ± 47.53 (^40^K) in Bq/kg, respectively. The activity concentration of potassium was found to be higher than the activities of thorium and uranium and revealed that the sediments samples from the study area is enriched with potassium which may be partly attributed to the chemical contents in the fertilizers rich in potassium applied to the soil in farming activities along the coastline and also due to the presence of loamy and clay sediments (El-Gamal et al., 2007; Reda et al., 2018; Amwaalanga et al., 2019). The average activity concentrations of ^238^U, ^232^Th and ^40^K exceeded the world average value of 35 Bq/kg, 30 Bq/kg and 400 Bq/kg respectively (UNSCEAR, 2000).

**Table 1:** Activity concentrations and radiological health risk parameters in shore sediment from Orange River.

Sample ID	Activity concentration (Bq/kg)			ADR (nGy/h)	AEDE (out) (mSv/y)	ELCR (10^−3^)
	U-238	Th-232	K-40			
O1	59.80 ± 9.21	46.43 ± 5.01	438.36 ± 62.21	57.90	0.71	2.49
O2	71.28 ± 8.02	54.77 ± 5.21	390.39 ±53.12	60.99	0.75	2.63
O3	80.13 ± 20.05	56.57 ± 5.32	404.69 ±60.01	64.76	0.79	2.77
O4	33.67 ± 2.92	37.24 ± 3.22	461.82 ± 63.41	52.55	0.64	2.24
O5	45.58 ± 3.94	37.15 ± 3.03	446.38 ± 63.02	50.22	0.62	2.17
O6	78.27 ± 9.04	63.00 ± 5.72	416.89 ± 60.42	71.05	0.87	3.05
O7	72.75 ± 8.11	58.96 ± 5.61	395.81 ± 53.33	67.69	0.83	2.91
O8	49.43 ± 4.01	44.23 ± 4.61	458.81 ± 63.40	58.20	0.71	2.49
O9	86.88 ± 21.12	67.01 ± 5.80	383.16 ± 48.02	73.17	0.90	3.15
O10	53.07 ± 7.04	38.27 ± 3.05	473.75 ± 63.52	53.48	0.66	2.31
O11	57.24 ± 8.21	42.65 ± 3.24	473.37 ± 63.44	56.39	0.69	2.42
O12	BDL	79.44 ± 6.01	451.97 ± 63.22	82.32	1.01	3.54
O13	59.27 ± 9.22	40.30 ± 3.95	330.39 ± 47.53	49.17	0.60	2.10
O14	74.09 ± 9.01	61.25 ± 5.72	409.48 ± 60.11	68.47	0.84	2.94
O15	59.53 ± 8.82	41.00 ± 3.97	437.83 ± 63.11	53.77	0.66	2.31
O16	77.26 ± 9.11	76.43 ± 6.71	400.22 ± 58.02	80.27	0.98	3.43
O17	67.01 ± 8.83	49.23 ± 4.76	398.24 ± 50.32	60.65	0.74	2.59
O18	114.27 ± 23.45	91.20 ± 9.22	397.89 ± 50.31	90.42	1.11	3.89
O19	70.47 ± 8.03	66.94 ± 5.71	411.92 ± 60.01	73.03	0.90	3.15
O20	59.21 ± 9.01	45.46 ± 4.65	358.42 ± 50.52	55.08	0.68	2.38
Minimum	BDL	37.15 ± 3.03	330.39 ± 47.53	49.17	0.60	2.10
Maximum	114.27 ± 23.45	91.20 ± 9.22	473.37 ± 63.44	90.42	1.11	3.89
Mean	63.46 ± 9.85	54.88 ±5.03	416.99 ± 57.85	63.98	0.78	2.73
WAV	35	30	400	59	0.07	0.29

BDL = below detection limit; WAV = World average value (UNSCEAR, 2000).

### 3.2 Radiological hazards indices

Table 1 displays the calculated values of absorbed dose rate (ADR), annual effective dose equivalent and the excess lifetime cancer risk (ELCR) obtained. The results showed that the values ranged from 49.17 to 90.42 nGy/h for ADR, 0.60–1.11 mSv/y for AEDE (outdoor) and 2.10 to 3.89 for ELCR respectively. The mean values obtained were found higher than the world recommended limits of 59 nGy/h, 0.07 mSv/y and 0.29 respectively for ADR, AEDE and ELCR (UNSCEAR, 2000). The implications of these high values which are above the critical limits is the possibility of the development of cancer in the region in the future.

### 3.3 Comparison of activity concentrations and absorbed dose rate with similar studies

**Figure 2:**
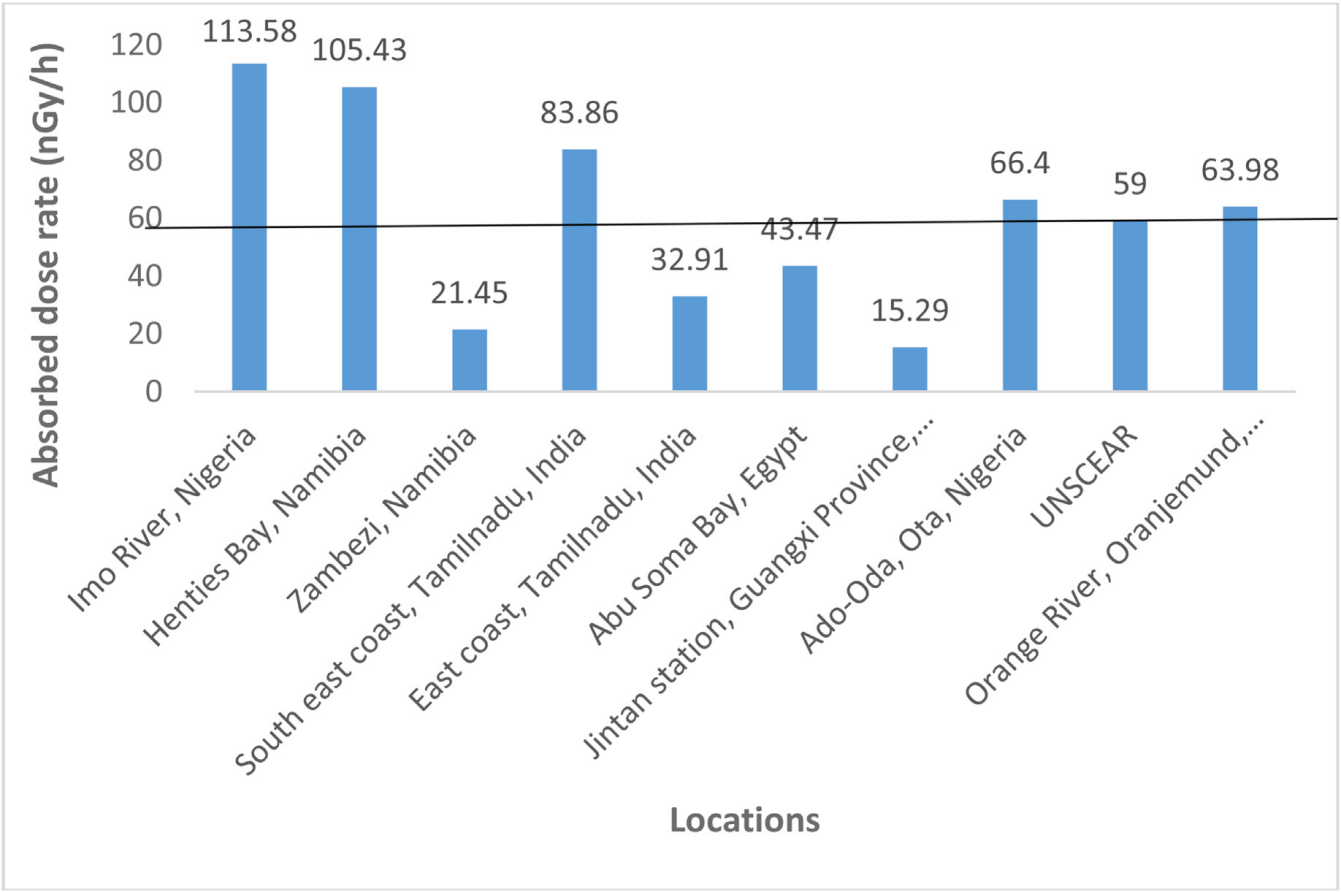
Comparison of absorbed dose rate of present study with others around the world.

The activity concentrations of ^238^U, ^232^Th and ^40^K assessed and the calculated absorbed dose rate in this present study compared with those in other areas is presented in Table 2 and Figure 2 respectively. The compared activity concentrations showed that the activity concentrations were above the recommended world values and the studies reported for China, Egypt, Nigeria as well as that of local monitoring done in Katima (Namibia) for ^238^U, World, China, Egypt, Henties Bay, Katima (Namibia), Malaysia and South Africa for ^232^Th and World, China, Egypt, Henties Bay (Namibia), Katima (Namibia), Malaysia and South Africa for ^40^K respectively. However, lower than the activities reported for Henties Bay (Namibia) for ^238^U, and Nigeria for ^232^Th and ^40^K respectively.

**Table 2:** Comparison of activity concentrations of ^238^U, ^232^Th and ^40^K from this study with other studies around the world.

S/N	Location	^238^U	^232^Th	^40^K	References
1.	World	35	30	400	UNSCEAR (2000)
2.	China	11.9	9.6	39.6	Xinming and Wuhui (2018)
3.	Egypt	22.7	11.6	93.0	Harb (2008)
4.	Nigeria	26.1	55.6	499.3	Omeje et al. (2021)
5.	Hentis Bay, Namibia	175.59	40.17	349.66	Onjefu et al. (2017)
6.	Katima, Namibia	18.91	15.58	79.17	Amwaalanga et al. (2019)
7.	Malaysia	ND	5.9	102	Khandaker et al. (2018)
8.	South Africa	ND	4.8	33.5	Newman et al. (2008)
9.	Orange River, Oranjemund, Namibia	63.46	54.88	416.99	**Present Study**

ND = No data.

Figure 2 shows a comparison of the absorbed dose rate of the present study with others reported around the world. The absorbed dose rate evaluated from this current work was found higher than those calculated in Katima (Namibia), India, Egypt, China and UNSCEAR recommended value while lower than those reported for Nigeria, and Henties Bay (Namibia) (Ononugbo et al., 2016; Onjefu et al., 2017; Amwaalanga et al., 2019; Havikrishnan et al., 2018; Sivakumar et al., 2014; Atef et al., 2018; Xinming and Wuhui, 2018; Omeje et al., 2021; UNSCEAR, 2000). The present study shows that the mean absorbed dose rate is 1.08 times higher than the world recommended limit. The level of gamma radiation is directly associated with the activity concentrations of radionuclides in the river shore sediments and cosmic rays (Taskin et al., 2009).

### 3.4 Statistical analysis

The statistical interpretations are presented in Table 3. The statistical data depicts the distributive pattern of radionuclides. The Skewness characterizes the degree of asymmetry of a distribution around its mean (Groeneveld and Meeden, 1984). In this study, the skewness of the activity concentrations of ^238^U and ^40^K were negative which indicates a distribution with an asymmetric tail extending towards values that are more negative while the activity of ^232^Th had positive skewness which shows that ^232^Th had distribution with an asymmetric tail extending towards values that are more positive.

**Table 3:** Basic Statistics of the measured data.

	^238^U	^232^Th	^40^K
Mean	63.4605	54.8765	416.9895
Standard Error	5.073736	3.466777	8.522267
Median	63.405	52	410.7
Standard Deviation	22.69044	15.5039	38.11274
Sample Variance	514.856	240.3709	1452.581
Kurtosis	3.087178	-0.07249	-0.09476
Skewness	-0.67437	0.802049	-0.3441
Range	114.27	54.05	143.36
Minimum	BDL	37.15	330.39
Maximum	114.27	91.2	473.75
Sum	1269.21	1097.53	8339.79
Count	20	20	20

The measure of probability distributive nature of a real-value random variable is known as the kurtosis. It is the measure of peakedness or flatness of a distribution when compared with a normal distribution. A positive kurtosis is indicative of a relative peaked distribution while a negative kurtosis represent a flat distribution (Sivakumar et al., 2014). In this present study, ^238^U have positive kurtosis while ^232^Th and ^40^K had negative kurtosis.

**Figure 3:**
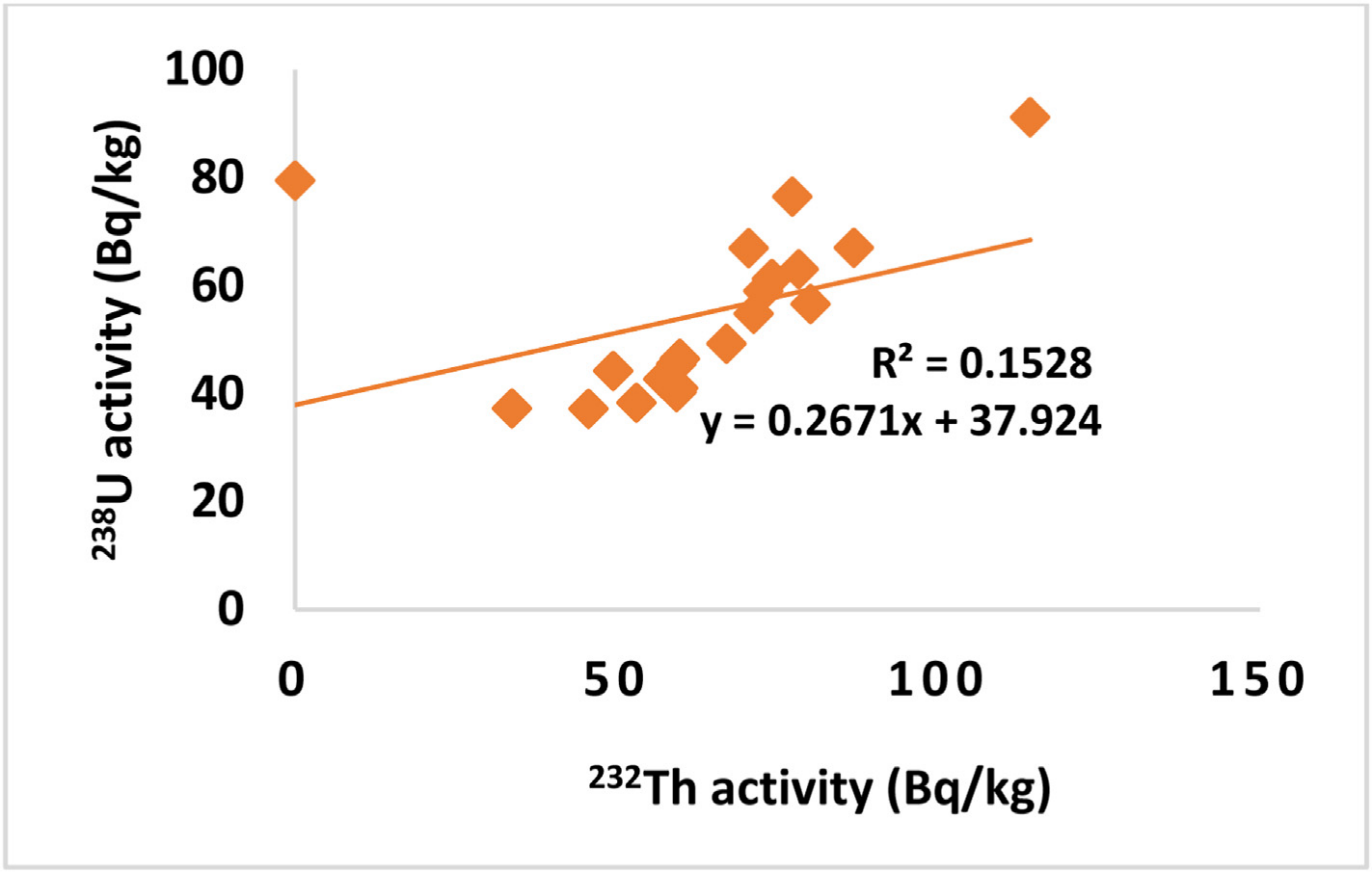
The correlation between ^238^U and ^232^Th.

**Figure 4:**
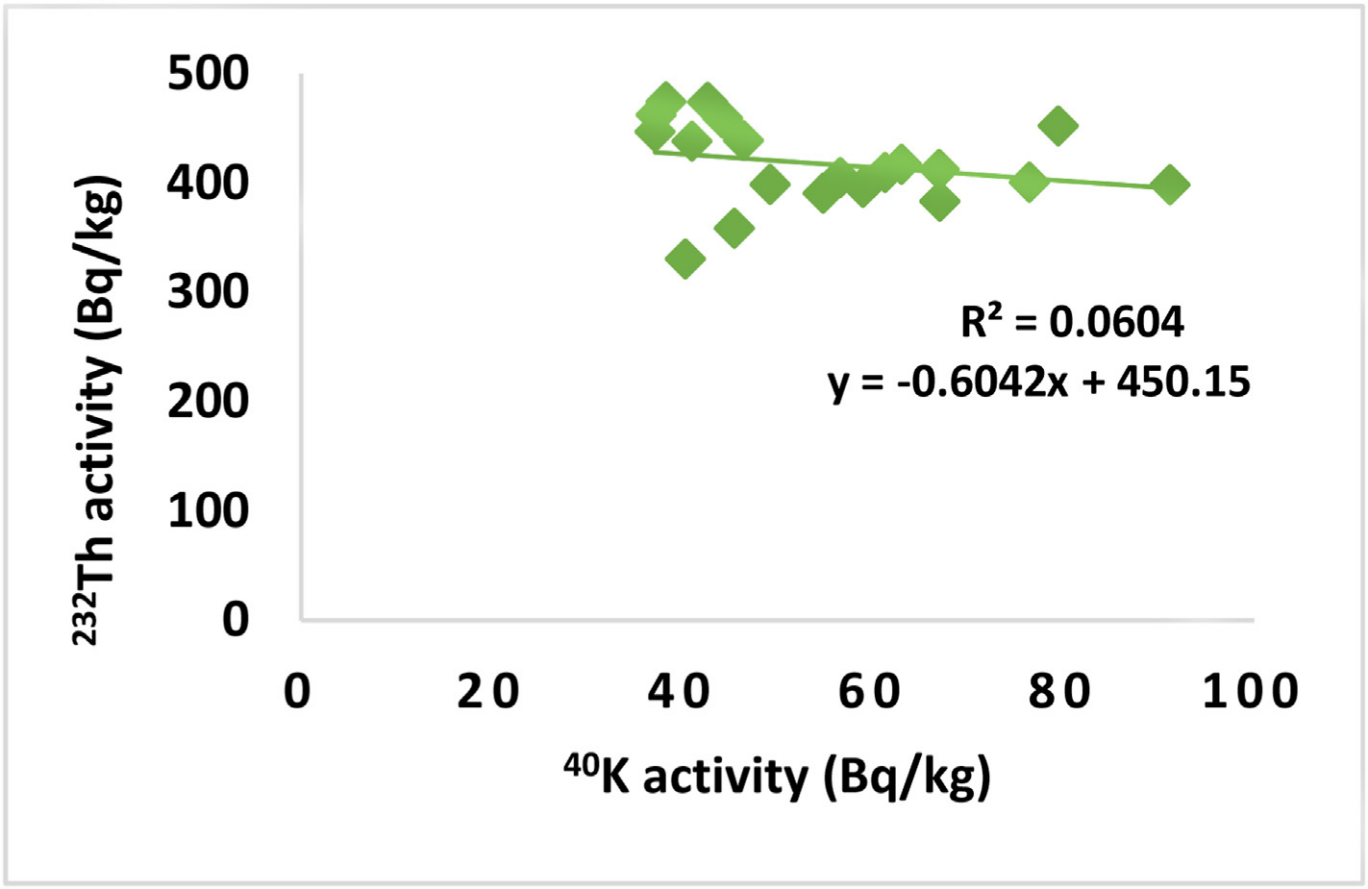
The correlation between ^232^Th and ^40^K.

**Figure 5:**
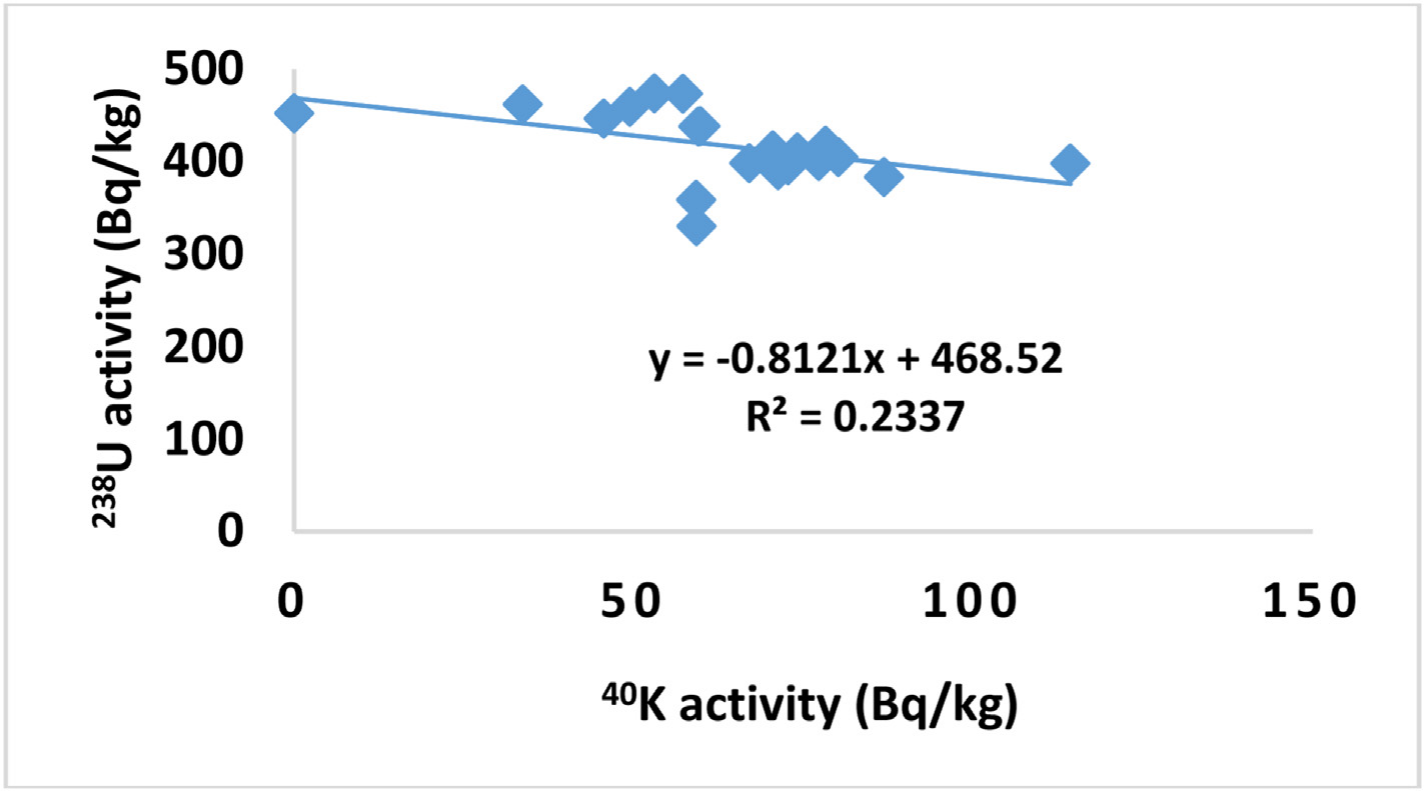
The correlation between ^238^U and ^40^K.

In order to test the correlations between the activity concentrations of ^238^U versus ^232^Th, ^232^Th versus ^40^K and ^238^U versus ^40^K, the activity obtained for ^238^U, ^232^Th and ^40^K were plotted as shown in Figures 3, 4, and 5 respectively. A weak positive correlation was found to exist between ^238^U and ^232^Th (R = 0.1528) which indicates a weak natural abundance of ^238^U and ^232^Th over period of times through their decay process while a weak negative correlation and insignificant correlation was found between ^238^U and ^40^K (R = 0.2337) and ^232^Th and ^40^K (R = 0.0604) which indicate the anomaly in potassium existence on earth crust due to human activity.

## 4 Conclusion

The activity concentrations of natural radionuclides from shore sediments along the coastline of the Orange River in Oranjemund, Namibia has been determined using a Canberra Gamma Spectrometry high purity germanium (HPGe) detector. The radionuclides detected were ^238^U, ^232^Th and ^40^K. The mean activity concentrations of ^238^U, ^232^Th and ^40^K were slightly higher when compared with worldwide average values. To quantify the effects associated with gamma rays from ^238^U, ^232^Th and ^40^K was calculated using the radiological parameters of absorbed dose rate, annual effective dose equivalent and excess lifetime cancer risk. The calculated values of the radiological parameters were found to be slightly above the recommended limits. The data obtained in this study may provide a general background concentration for the area and maybe useful in providing a guideline for future radiological investigation in the region. The radiological finding from the study may be used as a reference data for radionuclide monitoring and evaluation of natural radioactivity levels in the future.

## Declarations

### Author contribution statement

Onjefu SA, Kauluma ANI, Ejembi E, Zivuku M: Conceived and designed the experiments; Performed the experiments; Wrote the paper.

Hamunyela RH, Tyobeka MB: Analyzed and interpreted the data; Contributed reagents, materials, analysis tools or data.

### Funding statement

This research did not receive any specific grant from funding agencies in the public, commercial, or not-for-profit sectors.

### Data availability statement

Data included in article/supp. material/referenced in article.

### Declaration of interest’s statement

The authors declare no conflict of interest.

### Additional information

No additional information is available for this paper.
